# 3SAT on an all-to-all-connected CMOS Ising solver chip

**DOI:** 10.1038/s41598-024-60316-y

**Published:** 2024-05-10

**Authors:** Hüsrev Cılasun, Ziqing Zeng, Ramprasath S, Abhimanyu Kumar, Hao Lo, William Cho, William Moy, Chris H. Kim, Ulya R. Karpuzcu, Sachin S. Sapatnekar

**Affiliations:** 1 University of Minnesota, Minneapolis, USA; 2 Indian Institute of Technology Madras, Chennai, India

**Keywords:** Electrical and electronic engineering, Quantum information

## Abstract

This work solves 3SAT, a classical NP-complete problem, on a CMOS-based Ising hardware chip with all-to-all connectivity. The paper addresses practical issues in going from algorithms to hardware. It considers several degrees of freedom in mapping the 3SAT problem to the chip—using multiple Ising formulations for 3SAT; exploring multiple strategies for decomposing large problems into subproblems that can be accommodated on the Ising chip; and executing a sequence of these subproblems on CMOS hardware to obtain the solution to the larger problem. These are evaluated within a software framework, and the results are used to identify the most promising formulations and decomposition techniques. These best approaches are then mapped to the all-to-all hardware, and the performance of 3SAT is evaluated on the chip. Experimental data shows that the deployed decomposition and mapping strategies impact SAT solution quality: without our methods, the CMOS hardware cannot achieve 3SAT solutions on SATLIB benchmarks. Under the assumption of some hardware improvements, our chip-based 3SAT solver demonstrates a remarkable 250$\times$ acceleration compared to Tabu search in dwave-hybrid on a CPU.

## Introduction

Many combinatorial optimization problems (COPs), including NP-complete and NP-hard problems, can be solved using the quantum-inspired Ising model^1^, which originated from representations of magnetic interactions that settle to a minimum-energy state. These COPs can be written in Ising form via quadratic unconstrained binary optimization (QUBO) formulations, and then mapped to a network of coupled oscillators. As these oscillators settle to their minimum energy ground state, they solve the COP, potentially with better speed and energy than classical computers.

Many efforts have conceived or built Ising solvers in emerging technologies, e.g., quantum, spintronics, optics, phase change devices, NEMS, and ferroelectrics. However, these substrates have less desirable scaling properties compared to our time-domain coupled oscillator approach based on simple digital-like CMOS circuits; some require prohibitively expensive cooling to a few Kelvin. In contrast, CMOS-based Ising solvers^2–5^, which use coupled ring oscillators (ROs), can make Ising computation practical, delivering high speed, low power consumption, accuracy, high integration density, portability, and mass-manufacturability. A mixed-signal implementation has also been proposed^6^.

A limitation of many Ising machines is the limited connectivity between spin variables: D-Wave’s quantum-based solutions limit connectivity to 6–20 neighbors per oscillator^7^; even many CMOS-based solutions^2–4^ are limited to 4–8 nearest neighbors on a 2D oscillator mesh. The embedding problem of mapping the couplings in an Ising problem to this connectivity-limited structure requires spin replication: a six-variable problem with all-to-all interactions requires 18 spins on D-Wave’s Chimera and 30 spins on the King’s graph^5^. Replication weakens the strength of a spin, leading to suboptimal solutions^8^. Recent work^5^ breaks through these bottlenecks by implementing all-to-all (A2A) connectivity between 50 spins in a 65nm CMOS chip: its A2A connectivity makes it very powerful, equivalent to a locally connected architecture (e.g.,^2–4^) with thousands of spins^9^.

The problem of mapping COPs to an Ising hardware substrate is an open problem. *First,* multiple QUBO formulations are available for any COP, and some may perform better on hardware than others. *Second,* hardware engines operate under restrictions, e.g., the allowable values of coupling weights are limited. *Third,* since any hardware platform has limited capacity, large problems must be decomposed into smaller subproblems, and the decomposition strategy impacts solution quality.

To move Ising computing closer to reality, it is essential to provide a complete solution from algorithms to hardware execution. This work addresses these issues for 3SAT, a classical NP-complete problem^10^. The 3SAT problem was the “original” NP-complete problem^11^, and reduces to any other NP-complete problem through a polynomial-time transformation^12^. We examine multiple choices of QUBO formulation, decomposition, and mapping strategies, and report results on actual CMOS hardware: a 65nm Ising chip with A2A connectivity^5^. Depending on the formulation, the problem may require more or fewer spins, and more or fewer couplings; this systematic evaluation evaluates formulations to determine which delivers the best solution. Similarly, decomposition and mapping strategies can significantly impact solution quality^13^. Greedy, random and clustering-based decomposition algorithms are widely used in recent 3SAT or Ising solvers^14,15^.

The contributions of this paper include: (1) hardware-specific *evaluation of multiple mappings* from 3SAT to Ising models, (2) rigorous methods for *variable pruning* through spin removal optimization, (3) *scaling and local field oscillator optimizations* specifically for 3SAT, (4) three novel *decomposers* to break large problems to subproblems that fit on the hardware, (5) *hardware demonstration* of 3SAT benchmark instances on a CMOS Ising chip.

## Solving combinatorial problems on Ising machines

### QUBO/Ising problems and the underlying graph

A QUBO problem in *n* variables is formulated as minimizing a *Hamiltonian* objective function:

1 $$\begin{aligned} \min _{\textbf{x}} F(\textbf{x}) = \textbf{x}^T Q \textbf{x} = \sum _{i=1}^n Q_{ii} x_i + \sum _{i=1}^n \sum _{j=1, j \not = i}^n Q_{ij} x_i x_j \end{aligned}$$

where $\textbf{x} = [x_1, \ldots , x_n]^T \in \{ 0, 1 \}^n$ is a Boolean vector and $Q \in \mathbb {R}^{n \times n}$ is a real matrix; here, $Q_{ii}$ multiplies $x_i^2 = x_i$ for $x_i \in \{0, 1\}$. Using $x_i = (s_i + 1)/2$ to transform each Boolean variable $x_i$ to a spin variable $s_i \in \{-1, +1\}$, the Hamiltonian for the isomorphic **Ising formulation** is

2 $$\begin{aligned} \min _{\textbf{s}} F(\textbf{s}) = \sum _i h_i s_i + \sum _{i=1}^n \sum _{j=1, j \ne i}^n J_{ij}s_i s_j \end{aligned}$$

where $\textbf{s} = [s_1, \cdots s_n]^T \in \{+1,-1\}^n$, $J \in \mathbb {R}^{n \times n}$ is a real matrix, and $\textbf{h} = [h_1, \ldots , h_n]^T \in \mathbb {R}^n$ is a real vector, where $h_i = Q_{ii}/2 + \sum _{j=1}^n (Q_{ij} + Q_{ji})/4$ and $J_{ij}=Q_{ij}/4$.

The **graph representation** of the Ising formulation associates each variable $s_i$ with a vertex *i* with weight $h_i$, with coupled vertices *i* and *j* connected by an undirected edge of weight $(J_{ij}+J_{ji})$.

### A CMOS-based Ising hardware accelerator

**Figure 1:**
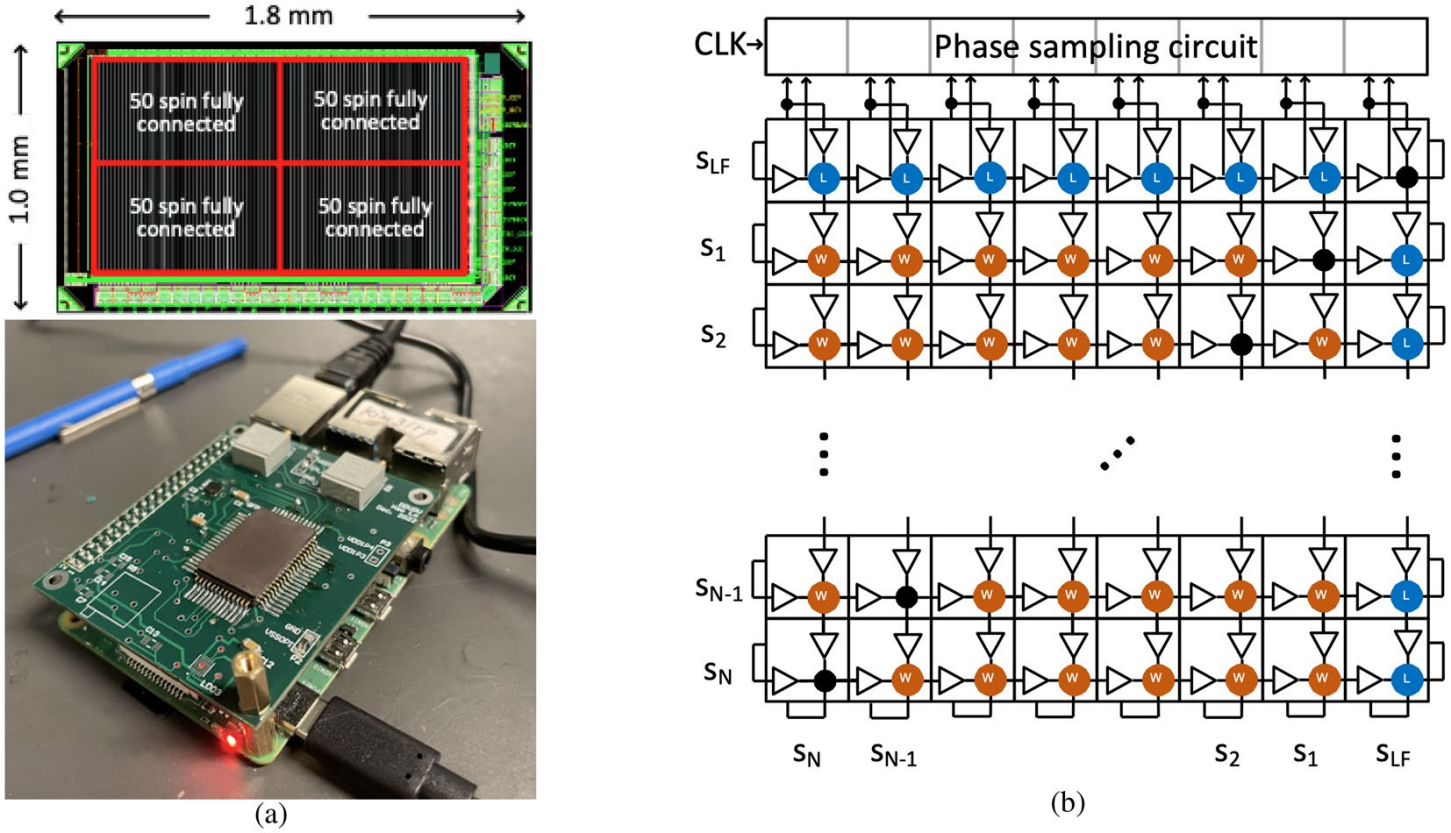
(**a**) Chip layout; chip soldered on a carrier board with a Raspberry Pi^5^. (**b**) All-to-all array of CMOS ROs^5^.

Figure 1 illustrates our hardware engine with an A2A architecture^5^, comprising (N$+1$) horizontal oscillators and (N$+1$) vertical oscillators. Each horizontal oscillator is short-circuited with the corresponding vertical oscillator on the diagonal, as shown by the black dots, so that the horizontal and vertical oscillators form a single physical oscillator carrying the same phase information. The spin variable associated with an oscillator corresponds to its phase. The paired oscillators denoted as s_LF_ are phase-locked and serve as the timing reference for the entire array, with spin value of $\mathrm{s_{LF}}$ fixed at $+1$; for other spins in the array whereas the spin values of $s_i$ are either $+1$ or $-1$, depending on whether the phase is the same as/opposite to $\mathrm{s_{LF}}$.

Setting $\mathrm{s_{LF}}=+1$ as a fixed reference, the coupling term $J_{i,\textrm{LF}} s_i \mathrm{s_{LF}} = J_{i,\textrm{LF}} s_i$. This becomes the local field term $h_i$ (= $J_{i,\textrm{LF}}$) in the Ising Hamiltonian equation. In Fig. 1, the coupling circuits along the bottom and right edges of the array, denoted as L, implement the $h_i$ weights. The intersection between spins $s_i$ and $s_j$, denoted as W, implement the coupling weights $J_{ij}$ between spins $s_i$ and $s_j$. The coupling circuits are implemented with transmission gates^5^. The weight $J_{ij}$ can be implemented by programming two locations – row *i*, column *j*, and row *j*, column *i* – in the array and the weight is the sum of weights in these locations. Since each coupling site can implement a weight of up to $\pm 7$, $J_{ij} \in \{-14,+14\}$.

The phase sampling block in Fig. 1 samples each RO 8 times in each cycle of the RO to generate an 8-bit binary result that is read out to determine the phase of the RO by majority voting^5^.

## Degrees of freedom in A2A hardware mapping

To solve a COP in Ising form on the A2A hardware of Sect. “A CMOS-based Ising hardware accelerator”, the process of mapping the Ising matrix and local field to the hardware must work within the hardware limitations. Since the coupling values $J_{ij}$ must be integers in the range $[-14,+14]$, smaller coupling weights of the problem may have to be **upscaled** while larger weights must be **downscaled** to lie in $[-14,14]$. This scaling step may result in non-integer values; these are rounded to an integer.

Conflicting considerations must be balanced during scaling: (1) The device accuracy is proportional to the coupling strength and therefore large scaling values are preferable. (2) If most of the weights are low in magnitude and only a few are high, the lower weights may be zeroed out during downscaling and integer rounding. To avoid this, some coupling weights may be scaled beyond the dynamic range and then clamped to the nearest extreme limit of the weight range. However, excessive scaling/clamping may alter the coupling matrix so greatly that its solution departs from that of the original problem.

A similar trade-off exists for the local field oscillators. The device allows an arbitrary number of spins to be configured as local field ROs (which implement $h_i$), while the remaining spins are configured to maintain pairwise coupling ($J_{ij}$ values). Increasing the number of Local Field Ring Oscillators (LFROs) can increase the dynamic range of the *h* coefficients: for example, a single LFRO can allow a coupling weight in the range $[-14,+14]$; if we perform *spin merging*, where two LFROs are used (and coupled tightly) to represent a single spin, a weight range of $[-56,56]$ is allowable. If the effective dynamic range of the $h_i$s is larger than that of the $J_{ij}$s, using more LFROs can allow higher coupling ranges with less truncation, improving accuracy; however, fewer spin variables will be available for problem mapping.

**Figure 2:**
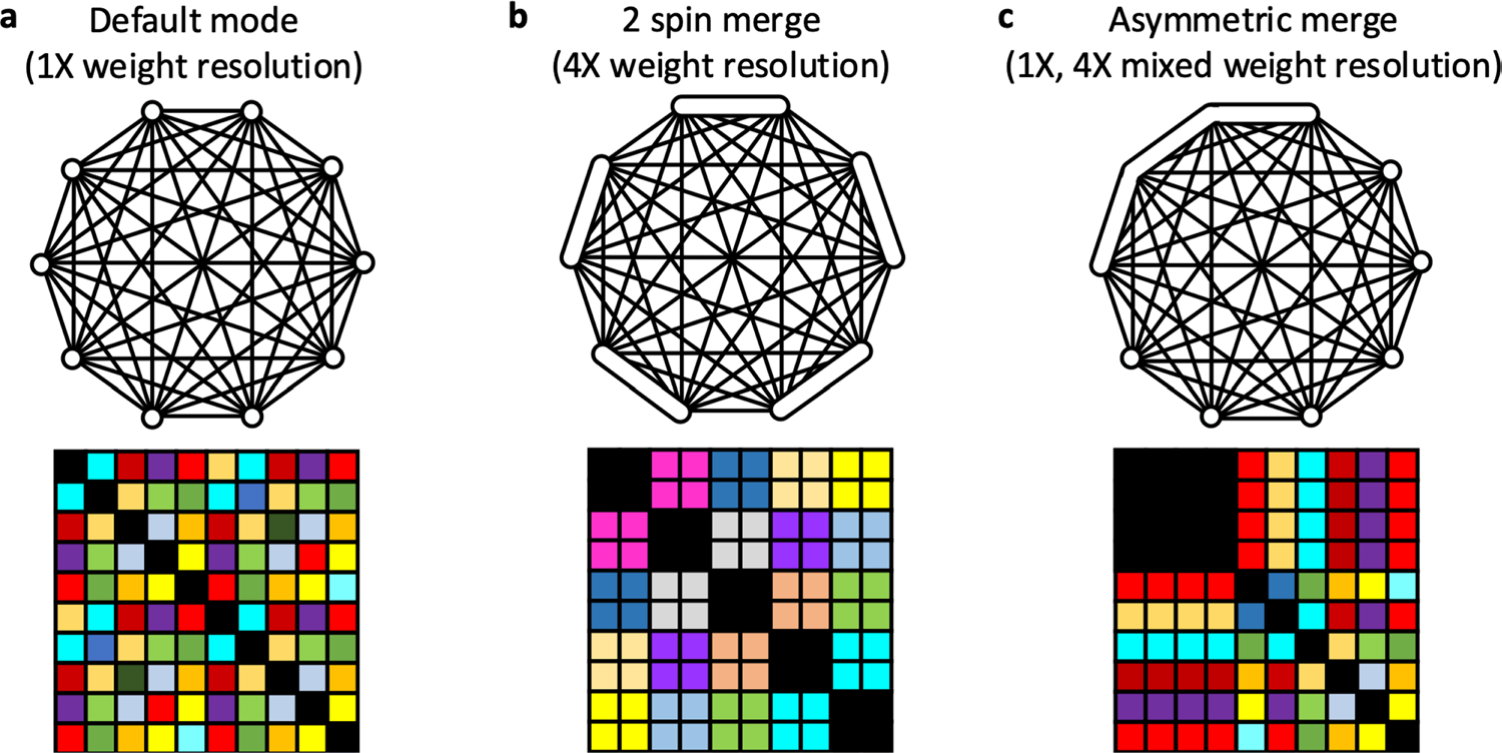
Illustrating spin-merging in the all-to-all array^5^.

We illustrate spin merging in Fig. 2^5^ where a 10-spin hardware example is configured to a 10-spin default mode, a 5-spin 4$\times$ resolution mode, and a 6-spin asymmetric resolution mode, respectively. The graph (upper row) and the corresponding hardware mapping (lower row) are shown for each configuration. In the latter, the black weight cells connect the vertical and horizontal oscillators, and the coupling cells are color-coded according to coupling strength. When two spins are merged (middle figure), coupling sites (each with weights up to $\pm 7$) lie on two $2 \times 2$ off-diagonal arrays. This allows coupling of $[-28,28]$ at each site and $J_{ij} \in [-56,+56]$. Thus, weight resolution can be traded off with the number of available spins.

## Formulating 3SAT for an Ising solver

The Boolean satisfiability problem seeks to find an assignment of input variables for which a Boolean function evaluates to logic 1. A 3SAT instance in Conjuctive Normal Form (CNF) is a conjunction of clauses, i.e., $f(x_1, \ldots , x_n) = C_1 \wedge C_2 \wedge \cdots \wedge C_m$, where $X = \{x_1, \ldots , x_n\}$ is a set of *n* Boolean variables. Each clause $C_i = l_1 \vee l_2 \vee l_3$ is a disjunction of at most three literals $l_1, l_2, l_3 \subset X \cup \lnot X$. A SAT formula *f* is *satisfiable* if there exists a set of Boolean assignments from $\{0,1\}$ on each variable in *X* that can be substituted such that $f(x_1, \ldots , x_n) = 1$; any combination of such variables, if it exists, is called a *satisfying assignment*.

Max-3SAT is a variant of the 3SAT problem that maximizes the number of satisfied clauses. If Max-3SAT satisfies all *N* clauses, then the corresponding 3SAT problem is satisfiable^16^. The Max-3SAT problem can be formulated in QUBO/Ising Hamiltonians using multiple formulations, described next in QUBO form; the spin formulation can be found as shown in Sect. “QUBO/Ising problems and the underlying graph”. The superscript against the name of each formulation provides the number of spins in the formulation as a function of *n* and *m*.

### The MIS^3m^ formulation

The maximal independent set (MIS) formulation^17^ establishes a graph by assigning a vertex (i.e., a QUBO variable) for each literal. For each clause, the three literals (vertices) are connected to each other, forming a “triangle” of edges. The literals of different clauses interact via conflict edges that connect any pair of vertices corresponding to literals $x_i$ and $\overline{x}_i$. For a problem with *m* clauses, this formulation requires 3*m* variables.

This formulation can be translated into QUBO form^18^ using up to 3*m* QUBO variables, one for each vertex in the graph. Given an instance $C_1 \wedge C_2 \wedge \dots \wedge C_m = (l_1 \vee l_2 \vee l_3) \wedge (l_4 \vee l_5 \vee l_6) \wedge \dots \wedge (l_{3m-2} \vee l_{3m-1} \vee l_{3m})$, the QUBO Hamiltonian is:

3 $$\begin{aligned} \sum _{i=1}^{3m} l_i + 2\left( \sum _{i=0}^{m-1} \sum _{j<k \in [1,3]} l_{3i+j} l_{3i+k} + \sum _{i,j|l_i=\lnot l_j} l_i l_j \right) \end{aligned}$$

By construction, the literals, $l_i$, have a many-to-one mapping to the original Boolean variables. If the literal values provide conflicting assignments to the Boolean variables, a majority vote is used to assign the value.

### An ILP^n+2m^ formulation

We propose a new integer linear program (ILP) formulation representing the $i^{\textrm{th}}$ clause by the Boolean inequality:

4 $$\begin{aligned} l_{3i+1} + l_{3i+2} + l_{3i+3} \ge 1 \end{aligned}$$

If the literal $l_j$ corresponds to variable $x_j$ in true form, then $l_j = x_j$; if negated, $l_j = 1- x_j$. The Max-3SAT problem is solved by finding a feasible solution to the ILP under these inequality constraints. Using a slack variable *s*, each inequality constraint is transformed to an equality constraint $l_{3i+1} + l_{3i+2} + l_{3i+3} - s - 1 = 0$, where $s \in \{0, 1, 2\}$, depending on whether one, two, or all three literals are 1. Encoding $s = 2s_{i,1} + s_{i,0}$, where $s_{i,1}$ and $s_{i,0}$ are binary variables, the equality constraint now contains all binary variables. This corresponds to minimizing the Hamiltonian:

5 $$\begin{aligned} \sum _{i=0}^{m-1} \left( l_{3i+1} + l_{3i+2} + l_{3i+3} - 2s_{i,1} - s_{i,0} - 1 \right) ^2 \end{aligned}$$

For example, clause $(x_a \vee x_b' \vee x_c)$ is encoded as $x_a + (1 - x_b) + x_c - 2s_1 - s_0 - 1 = 0$, and its contribution to the Hamiltonian is the square of the left hand side. An instance with *n* variables and *m* clauses has $n+2m$ QUBO variables, including two ancillary slack variables for each of *m* clauses. Since typically, $n < m$, ILP^n+2m^ has fewer Ising variables than MIS^3m^, but the range of weights is higher and the connectivity is denser. Unlike the MIS^3m^ formulation, there is a 1–1 correspondence between the first *n* QUBO/Ising variables and the Boolean Max-3SAT variables, and no contradictions need to be resolved after solution.

### The Chancellor^n+m^ formulation

The Chancellor formulation^19^ maps an *n*-variable *m*-clause instance using $n+m$ QUBO/Ising variables, with a 1–1 correspondence between the *n* SAT variables and the first *n* QUBO/Ising variables, and one ancillary variable for each of the *m* clauses. Denoting the SAT variables as $x_1, \ldots , x_n$ and the ancillary variables as $x_{n+1}, \ldots , x_{n+m}$, the overall Hamiltonian is:

6 $$\begin{aligned} { \sum _{i=0}^{m-1} \left( -(l_{n+i}+1)(l_{3i}+l_{3i+1}+l_{3i+2} )+2l_{n+i}+\sum _{j<k \in [0,2]}l_{3i+j}l_{3i+k}\right) } \end{aligned}$$

As in ILP^n+2m^, for a literal $l_j$ in true form, $l_j = x_j$; else $l_j = 1- x_j$.

### The Nüßlein^2n+m^ formulation

The Nüßlein^2n+m^ formulation^20^ maximizes the number of satisfied clauses by making the Hamiltonian equal to the negative of the number of the satisfied clauses. For this purpose, a dual of each of the *n* original variables is designated to obtain the variable pairs $x_i, x_{i+1}$ that correspond to the $i^{\rm th}$ 3SAT variable. These are one-hot-encoded to 10 if the 3SAT variable is true, and 01 if it is false. Additionally, one ancillary variable is designated for each of *m* clauses, leading to $2n+m$ variables. The corresponding QUBO Hamiltonian is:

7 $$\begin{aligned} {-}\sum _{i=1}^{2n} R(x_i) x_i^2 + 2 \sum _{i=2n+1}^{2n+m} x_i^2 + \sum _{i=1 |(i\mod 2) \not = 0}^{2n-1} (m+1) x_i x_{i+1} + \sum _{i=1}^{2n} \sum _{j=1}^{2n} R(x_i,x_j) x_i x_j - \sum _{i=1}^{2n} \sum _{j=2n|x_i \in c_{j-2n}}^{2n+m} x_i x_j \end{aligned}$$

where $R(x_i)$ is the number of clauses that contain $x_i$, and $R(x_i,x_j)$ is similarly defined as the number of clauses such that contain both $x_i$ and $x_j$. This Hamiltonian aims to make the energy contribution of each satisfied clause $-1$ (and each unsatisfied clause 0). The formulation rewards each variable if it satisfies a clause (first term), and the local field coefficient of the ancillary variable of each clause is assigned to 2 (second term). Assignments of both a Boolean variable and its complement to 1 are penalized to ensure consistency (third term); the case where both are 0 effectively means that the variable is a don’t care.

### The Nüßlein^n+m^ formulation

Although Chancellor^n+m^ requires fewer QUBO variables than MIS^3m^ and Nüßlein^2n+m^, it uses relatively more coupling weights. The Nüßlein^n+m^ formulation^20^ has a lower number of nonzero couplings between QUBO/Ising variables. Nüßlein^n+m^ uses four different clause literal negation patterns^20^ for Max-3SAT, where each pattern corresponds to the number of negated literals in the clauses. The formulation then consists of constructing (or *updating*) the Hamiltonian, clause by clause. Each pattern ensures that post(pre)-update Hamiltonian $H^{+}$($H^{*}$) satisfies $H^{+}=H^{*}$ if the immediate clause is satisfied, and $H^{+}=H^{*}+1$ otherwise. Based on this rule and the negation pattern of each clause, the Hamiltonian is updated iteratively for each clause.

In summary, these formulations each possess distinct features, such as the number of spins in Hamiltonian, the density of nonzero coupling, and the range of coupling values. We will compare these on benchmark testcases in Sect. “Experimental setup and metrics”.

## Implementation workflows

### Overview of our hybrid solver approach

**Figure 3:**
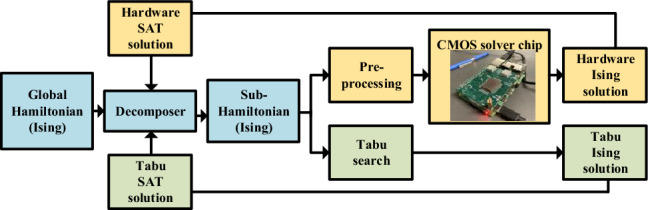
Workflow of our Hybrid SAT solver.

Any hardware solver has a limited number of spins, and large problems must be decomposed into smaller subproblems that can fit on the hardware, and solved iteratively until the ground state is found. The qbsolv^15^ engine performs a similar decomposition, purely in software, optimizing a large QUBO problem by solving a series of sub-QUBO problems using local Tabu search. Figure 3 shows two workflows for 3SAT solution and evaluation: a hybrid hardware-based flow and a purely software-based flow. The software flow is based on qbsolv, but augmented with new methods that we propose for problem decomposition.

The software workflow emulates the A2A hardware results, but is free of the nonidealities and noise in the hardware, and thus represents the best achievable results for the hardware-based flow. We use it to evaluate the effectiveness of various Ising formulations and decomposer schemes, and prune the list of candidates implemented on the Ising hardware. This pruning is useful due to the limitations of the prototype Ising chip, which focuses on optimizing the A2A engine, but currently has slow input/output (I/O); this I/O bottleneck can be easily resolved using standard techniques in future versions of the chip.

In the figure, the blue boxes are performed on a classical computer and are common to both workflows. We first transform the 3SAT problem into a global Hamiltonian (Ising) formulation (Sect. “QUBO/Ising problems and the underlying graph”). The size of the Hamiltonian depends on the 3SAT problem and the specific formulation used to map 3SAT to QUBO (Sect. “Formulating 3SAT for an Ising solver”). To map the Hamiltonian to hardware, we use a decomposer (Sect. “Decomposers”) to generate an Ising sub-Hamiltonian that can fit the dimensions of the RO array: on our A2A hardware, this allows at most 49 spins, including the reference.

For the **hardware** workflow (yellow boxes in Fig. 3), this sub-Hamiltonian is preprocessed (Sect. “Preprocessing for RO array”), and the Ising weights are programmed on to the chip. The spin states on the chip are sampled to determine the phase for each RO using majority voting. The **software** workflow (green boxes in Fig. 3) uses Tabu search^21^ to solve the same sub-Hamiltonian.

For both hardware-based and software-based evaluation, the decomposer sequentially sends each sub-Hamiltonian for processing. This terminates when all clauses are satisfied, i.e., “all-SAT” is achieved, or if a predefined iteration limit is reached.

### Decomposers

We study five decomposers: the last three are developed by us.

**Energy impact decomposer.** This decomposer, the qbsolv default, arranges spins in ascending order based on their flip energy (i.e., the energy difference when spin $s_{i}$ is flipped to $-s_{i}$), and greedily selects spins with the highest flip energy to construct the sub-Hamiltonian. Such a greedy algorithm is liable to be trapped in a local minimum.

**Random decomposer.** This decomposer randomly selects spins from the global Hamiltonian to form the sub-Hamiltonian. The randomness in the selection process helps the algorithm escape from local minima.

**Pseudorandom decomposer.** Our heuristic scheme continually reshuffles and shifts the variable order, selecting the first *S* variables at each iteration for the sub-Hamiltonian solver, where *S* represents the number of spins supported by the hardware. The pseudorandom decomposer ensures diversity among the generated sub-Hamiltonians.

**BFS decomposer.** This scheme creates a cluster around a randomly-selected source vertex in the Ising graph. A breadth-first search (BFS) in the graph starts from the source, adding neighbors in randomized order until the capacity is reached.

**SAT decomposer.** This clustering-based decomposer randomly selects one clause at each step and adds all spins related to the selected clause and its involved variables to the sub-Hamiltonian.

### Preprocessing for RO array

For the hardware workflow, each decomposed sub-Hamiltonian is adapted to satisfy the restrictions imposed by the Ising chip:

1. Sub-Hamiltonian coupling weights must be restricted to integers in the interval $[-14,+14]$.
2. Coupling values should be as large as possible: empirically, device accuracy improves with coupling strength.

We translate the sub-Hamiltonian from the decomposer to a hardware-compatible coupling matrix, using the following methods:

**Mapping**: Increasing the number of LFROs and using spin merging for the local field increases the dynamic range of the *h* coefficients (Sect. “Degrees of freedom in A2A hardware mapping”). In Sect. “Experimental setup and metrics”, we sweep the number of LFROs to choose an optimal number of LFROs to be merged.

**Removing spin variables from the sub-Hamiltonian**: A large local field on a spin variable can force the variable to a fixed value. The contribution of a spin $s_i$ on the Ising Hamiltonian is $H(s_i) = h_i s_i +\sum _{j=1, j \not = i}^n J_{ij} s_i s_j$, and the flip energy is

8 $$\begin{aligned} H_{flip}(s_i) = H(s_i=+1) - H(s_i=-1) = 2 \left( h_i + \sum _{j=1, j \ne i}^n J_{ij} s_j \right) \ge 2 \left( h_i - \sum _{j=1, j \ne i}^n |J_{ij}|\right) \end{aligned}$$

The last inequality comes from the relation, $\sum _{j=1, j \ne i}^n J_{ij} s_j \ge - \sum _{j=1, j \ne i}^n |J_{ij}|$. For $h_i > 0$, if $h_i > \sum _{j=1, j \ne i}^n |J_{ij}|$, then $H_{flip}(s_i) > 0$ regardless of the choice of the other spins. Thus, $H(s_i = +1) > H(s_i = -1)$, i.e., a minimum Hamiltonian will force $s_i = -1$.

Similarly, using the upper bound $\sum _{j=1, j \ne i}^n |J_{ij}|$ on the second term in (8), $H_{flip}(s_i) \le 2 (h_i + \sum _{j=1, j \not = i}^n |J_{ij}|) < 0$. Therefore, if $h_i < -\sum _{j=1, j \not = i}^n |J_{ij}|$ < 0, minimizing the Hamiltonian forces $s_i = +1$.

Together, these cases show that **if **$h_i$**is very negative [very positive]**, $s_i = +1$ [$s_i = -1$]**at the minimum**, and the corresponding spin variable can be removed from the Hamiltonian. Since the weights in the hardware are limited to $[-14,+14]$, this serendipitously allows us to remove large/small weights, at no loss of accuracy.

**Figure 4:**
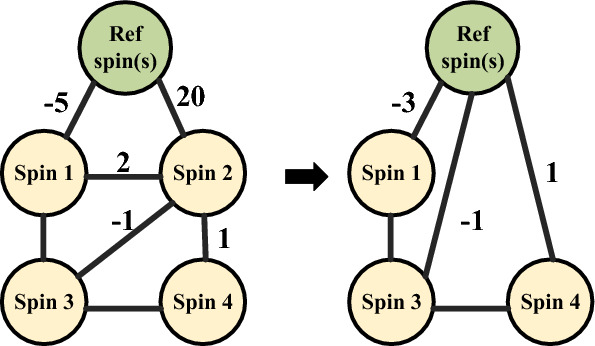
An example where spin 2 can be removed (set to the reference spin).

In practice, we use the criterion $|h_i|> N \times \max _j |J_{ij}|$, for a tuned value of *N*, and find it to be effective in identifying spins that could be removed, empirically without loss of optimality. In Fig. 4, the coupling between spin 2 and reference spin (Ref) $h_2=20$ is much larger than $\max _j J_{2j} = 2$. Therefore, spin 2 is removed from the Hamiltonian. All couplings, $J_{2j}$, are transferred into couplings with the reference spin ($h_j$), and spin 2 is set to $+1$.

**Scaling**: Since large coupling values are preferable for device accuracy, and minimizing $F(\textbf{s})$ in (2) is identical to minimizing $kF(\textbf{s})$ for any scalar *k*, we can scale the $h_i$ and $J_{ij}$ values up as long as the maximum value lies in the range $[-14,+14]$.

**Truncation**: Scaled coupling values beyond $\pm 14$ are truncated by clamping them to $+14$ or $-14$.

In summary, insights from our experiments on hardware implementation include: (1) Scaling up weights for sub-Hamiltonians with small coupling values improves performance. (2) The number of LFROs and the scaling factor should be considered together to find an optimal combination. (3) For Ising problems with more spins and similar coupling values, more LFROs may improve the performance after decomposition. (4) Excessive scaling, and too many LFROs, lead to a decrease in overall performance. In Sect. “Experimental setup and metrics”, we experimentally obtain a methodology for the mapping algorithm.

## Experimental setup and metrics

We present three experiments in this section: (1) the software workflow, focusing on Hamiltonian formulations and choices for the decomposer; (2) a hardware sub-Hamiltonian workflow test, focusing on preprocessing; (3) using the optimal configuration from the first two experiments to solve 3SAT benchmarks using our complete workflow.

We use 10 benchmarks from the SATLIB uf20-91 suite^22^. All benchmarks are satisfiable and each includes 20 variables/91 clauses. The clauses-to-variables ratio, $m/n = 4.55$ represents problems that lie close to the *phase transition region*^23^ where the hardest SAT problems lie. We use the following metrics:

**Iterations**: Each *iteration* of our workflow generates a decomposed sub-Hamiltonian, sent to the Ising solver in an inner loop. The 3SAT problem is solved over multiple sub-Hamiltonian solutions.

**Repeats**: The 3SAT/Hamiltonian solution is *repeated* many times in the outer loop of our workflow, thus reducing the impact of the random initial states or noise effects.

**All-SAT ratio**: This quality metric is the number of repeats that find an all-SAT solution, divided by the total number of repeats.

**Energy ratio**: This is the ratio of the sub-Hamiltonian energy of the current solution and the ground state (from the software workflow), and indicates the accuracy of the sub-Hamiltonian solution.

**Figure 5:**
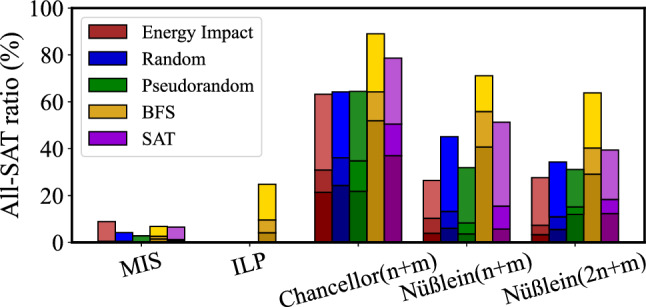
Evaluation of the five formulations (MIS, ILP, Chancellor^n+m^, Nüßlein^n+m^, Nüßlein^2n+m^) and decomposers (energy impact, random, pseudorandom, BFS, SAT: the pseudorandom, BFS, and SAT decomposers are developed in this work).

In Fig. 5, we explore the multiple Ising formulations of the 3SAT problem, and multiple decomposition strategies using the software workflow. We show the average All-SAT ratio out of 100 repeats for the first 10 instances in the uf20-91 benchmarks at the end of 50, 100, and 500 iterations, as denoted with darker-to-lighter tones from bottom to top in each bar. *On average, the best performance comes from the Chancellor*^n+m^* formulation and the BFS decomposer.*

**Figure 6:**
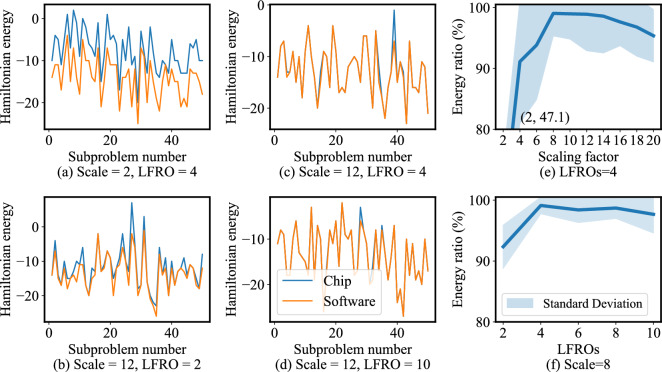
Software and chip Hamiltonian energy for the uf20-91/01 instance (**a**) Energy for Scale 2 with 4 LFROs, (**b**) Energy for Scale 12 with 2 LFROs, (**c**) Energy for Scale 12 with 2 LFROs, and (**d**) Energy for Scale 12 with 10 LFROs, (**e**) Energy ratio for scaling (the point (2,47.1) is listed but not shown in the plot: adding this point would make it harder to see the variation for higher values of Scaling factor), (**f**) Energy ratio for LFROs. In (**e**) and (**f**), the solid line is the mean of the Hamiltonian energy, and the shaded region marks one standard deviation from the mean, over all repeats.

For sub-Hamiltonian hardware mapping, we show the effects of scaling and mapping (i.e., number of LFROs) in Fig. 6. The Hamiltonian from the software (ideal) and hardware workflows, for each decomposed subproblem of the uf20-91/01 benchmark, are provided in Fig. 6(a) for a scaling factor of 2 (all couplings multiplied by 2) with 4 LFROs, and in Fig. 6(b)–(d) for scaling by 12, with 2, 4, and 10 LFROs, respectively; scaled values beyond $\pm 14$ are clamped using truncation. Figure 6(a) shows a significant discrepancy between the ideal and hardware Hamiltonian energies, while in Fig. 6(c) with higher scaling and the same number of LFROs, the hardware follows the software much more closely due to the stronger coupling of spins, thus reinforcing the empirical observation that stronger coupling improves chip accuracy. At the higher scaling factor of 12, 2 LFROs in Fig. 6(b) provide better accuracy than the Scale 2 case. Moreover, 4 and 10 LFROs in Fig. 6(c) and (d), respectively, provide progressive improvements, as they provide a larger dynamic range for the *h* coefficients, requiring fewer truncations. *Appropriate selection of scaling factors and the LFROs thus bring hardware solutions closer to the ideal ground state.*

We examine the statistics of subproblem accuracy based on the energy ratio. In Fig. 6(e) and (f), we, respectively, vary the scaling factor, fixing the number LFROs to 4; and vary the number of LFROs, fixing the scaling factor to 8. We show results on the uf20-91-01–uf20-91-10 SAT benchmarks for 50 iterations. Both scaling factor and LFRO optimizations affect the number of coupling weights that are truncated due to the insufficient dynamic range. A scaling factor of 2 provides the fewest truncated variables, but subproblem accuracy is low due to insufficient coupling strength. As the scaling factor increases, the subproblem accuracy improves despite more and more variables being truncated, showing the tradeoff between the dynamic range and the coupling strength. *Thus, the energy landscape transformation due to insufficient dynamic range affects the subproblem solution accuracy in such a way that the number of truncated coefficients is correlated with the accuracy loss. Overall, a scaling factor of 8 alongside 4 LFROs provides the best subproblem accuracy (Energy ratio).*

In general, identifying a universal hardware mapping algorithm proves challenging due to the preference for high coupling values and the intricate balance between dynamic range and coupling strength. Therefore, we limit the optimization of the mapping algorithm to selected 3SAT benchmarks and tune our hardware preprocessing accordingly. It’s important to note that the optimality of the preprocessing algorithm utilized here may not extend directly to other problems.

**Figure 7:**
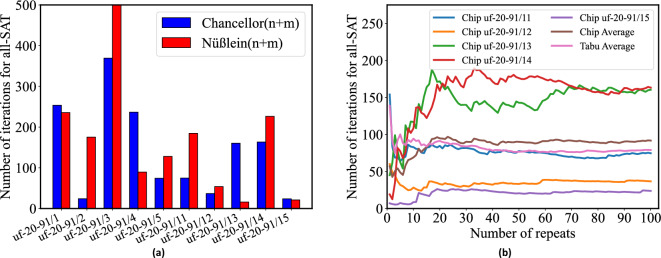
Number of iterations to find All-SAT for hardware test on the Ising chip for (**a**) Average number of iterations for uf20-91/(01-05, 11-15) for Chancellor^n+m^ and Nüßlein^n+m^. (**b**) Average number of iterations with repeats for uf20-91/(11–15) in the formulation of Chancellor^n+m^ with solver chip and average number of iterations with Tabu search.

Based on Fig. 5, we select the two formulations with the best average performance (Chancellor^n+m^ and Nüßlein^n+m^), and the best decomposer (BFS) for our hardware solver. We use instances 01–05 of uf20-91, used previously, and an orthogonal set, instances 11–15 of uf20-91, which represent unseen data. Over 100 repeats for each benchmark, with a time-out at 500 iterations, we plot the average number of iterations to reach all-SAT in Fig. 7a, overall 10 instances. Chancellor^n+m^ generally achieves all-SAT in fewer iterations than Nüßlein^n+m^, with a few exceptions, e.g., uf20-91/13. However, in one case (uf20-91/03), where the average number of iterations exceeds the time-out limit of 500, Nüßlein^n+m^ is unable to achieve all-SAT. Figure 7b shows the evolution of the average number of iterations over 100 repeats for instances 11–15, settling to the steady-state value in Fig. 7a. Specifically, over 100 repeats, the average number of iterations is 91.8 for the chip and 79 for the software Tabu search; the difference is due to the accuracy loss in going to the hardware.

**Table 1:** Runtime analysis for our hardware solution.

	Runtime per unit operation	Multiplier for previous column	Number of samples	Runtime
Input	$1.25\times 10^{-3}\upmu$s/bit	9604 bits	1	12.0$\upmu$s
RO relaxation	(1/26MHz) s/cycle	40 cycles	100	153.8$\upmu$s
Output	$1.25\times 10^{-3}\upmu$s/bit	49 bits	100	6.1$\upmu$s
Overall runtime				171.9$\upmu$s

**Runtime analysis.** A runtime analysis of our Hybrid solver is illustrated in Table 1, assuming 100 samples on the Ising solver chip; the sample with the lowest Hamiltonian value is selected. As an academic demonstrator focuses on optimizing the A2A array, with a focus on optimizing the all-to-all array, the Ising chip has several limitations:

1. The existing low IO bandwidth can be improved (800Mbps can be achieved with 8-bit parallel IO at 100Mbps each).
2. Majority voting (Sect. “A CMOS-based Ising hardware accelerator”) can be performed on-chip, reducing output data by 8$\times$, from 8 bits/spin to 1 bit/spin.
3. Decomposition can be performed on-chip where the indices are precomputed.
4. The Hamiltonian computation to check for convergence is currently performed off-chip, using the Ising energy computation function in the dimod^24^ package on an Intel Xeon 4114 CPU. This computation can also easily be performed in hardware; in fact, the computation can be overlapped with the next Ising solution, and it incurs no additional latency.

All of these are relatively simple extensions, and the only reason that they are not on the chip already is it is an academic project, limited by the number of students who can work on tape-outs, and the hardware focus has been on enhancing the core A2A engine. To project the true power of this hardware computational model, we use the settling time of the current version of the Ising chip, and project the total runtime numbers under the assumption that the above four improvements are made. Under these assumptions, the overall runtime is 171.9$\upmu$s per iteration, taking 100 samples in each iteration. Our runtime estimate does not include the decomposition runtime. Our random source-based BFS decomposer (Sect. “Decomposers”) has no dependency on previous iterations, and search indices can be precomputed. Moreover, the overhead of updating our $45$ sub-Hamiltonian with 10% nonzero density in hardware implementation is projected to only take a few hundred cycles (see Supplementary Materials), which is negligible.

**Table 2:** Runtime and energy consumption comparison between Tabu, WalkSAT, and the Ising chip.

Benchmark	Runtime (ms)			Energy (mJ)		
	Tabu	WalkSAT	Chip	Tabu	WalkSAT	Chip
uf20-91-11	4322	2.351	12.83	367,370	199.8	0.128
uf20-91-12	1596	2.337	6.30	135,660	198.6	0.063
uf20-91-13	5095	2.506	27.58	50,950	213.0	0.276
uf20-91-14	7148	2.470	28.07	433,075	210.0	0.281
uf20-91-15	1591	2.377	4.09	607,580	202.0	0.041

Based on the iteration counts presented in Fig. 7, Table 2 shows the runtime and comparison among Tabu search, our chip-based solution, and WalkSAT^25^, on a set of benchmarks. Compared to the software Tabu search based on D-Wave Hybrid^26^, the speedup of our solver ranges from 185$\times$–389$\times$, with an average speedup of 250$\times$. Given the power is 10 mW for our 10% density problems^5^, the energy is improved by orders of magnitude. The runtimes of WalkSAT are better than those of our current solver, but since WalkSAT is run on a CPU, its power dissipation is considerably higher than the 10 mW power for our chip. As a result, the energy consumption of our chip-based solver is seen to be much less than WalkSAT.

## Conclusion

This work solves 3SAT on a CMOS-based Ising chip, addressing degrees of freedom in problem formulation (the Chancellor^n+m^ formulation performed best on average) and problem decomposition (BFS decomposition performed best on average), as well as hardware mapping strategies for the Ising problem that extract the best performance from the chip. To the best of our knowledge, this is the first comprehensive exploration of these issues, paving the way towards bringing Ising computation to the mainstream through algorithm mapping on a mass-manufacturable CMOS chip. After optimization of the chip, we project that our chip-based 3SAT solver can achieve 250$\times$ speedup than the Dwave-Hybrid^26^ software-based solver with the power of 10mW^5^. Our work is currently based on randomly-generated SATLIB benchmarks and it has been observed^27^ that heuristic SAT solvers have better performance over industrial real-work benchmarks over randomly-generated benchmarks. We intend to investigate this issue in future work, and scaling up the solution to larger problems using multiple Ising solver cores.

### Supplementary Information

Supplementary Information.

## Data Availability

The datasets used and/or analyzed during the current study are available from the corresponding author on reasonable request.
