# Supplementary material for: 3SAT on an all-to-all-connected CMOS Ising solver chip
Source: Sci Rep. 2024 May 10;14:10757. doi: 10.1038/s41598-024-60316-y (PMC11087575; doi:10.1038/s41598-024-60316-y)
Supplement: Supplementary file 1 — Supplementary Information. [file 41598_2024_60316_MOESM1_ESM.pdf]

Supplementary Materials: 3SAT on an All-to-All-Connected CMOS Ising Solver Chip

1 Chip Specifications

The CMOS Ising chip on which our experiments are performed has 49 coupled ring oscillators in an all-to-all topology, built in a TSMC 65nm technology. The dynamic range of the J_{ij} and h_i weights ranges from -14 to $+14$, including 0. The parameters of the CMOS Ising solver chip on which our results are generated are listed in Table **??**.

Unique features of this chip are: the use of CMOS technology, which provides orders-of-magnitude lower power dissipation than non-CMOS substrates that have to be cooled to very low temperatures[1]; a proven mass-manufacturable hardware substrate with proven reliability, unlike more exotic emerging technologies[2–8]; and the high connection versatility due to all-to-all connectivity between spins, which has not been available to prior CMOS-based solutions[9–12].

Table S1. Specifications of the CMOS Ising chip.

Technology	TSMC 65nm
Topology	All-to-all
Spin count	49
Weights (J_{ij}, h_i)	$\{-14, \cdots, +14\}$ (29 levels)
Maximum power	0.12W @1.3V
Ising core area	0.28mm^2
Oscillation frequency	26MHz
Relaxation time	40 cycles

2 Pseudocode of our Workflow

Software algorithm. The full software algorithm (referred to as "qbsolv" in the paper) is as described in the pseudocode in Algorithm 1. Note that this software algorithm, described by the green path in Fig. 3 of the manuscript, does not use the chip at all, and is performed purely on the host.

- Starting from the Ising formulation, we first initialize all spins to -1 before starting the decomposition.
- In each iteration, we use a decomposer to choose which spins are to be included in the subproblem that is solved during the iteration. The software solver uses Tabu search to solve the subproblem and update the corresponding spins, as shown by the green path in Fig. 3.
- For each current state of the Tabu search, we check to see whether we have a solution that satisfies all clauses. If so, the solution is marked "all-SAT" and the iteration is concluded; otherwise, we generate the next subproblem and continue the process until either all-SAT is achieved, or the timeout condition is met.
- We repeat the above process for the specified number of repeats and report the all-SAT rate.

Hardware algorithm. The hardware algorithm, which corresponds to the yellow path in Fig. 3 in the manuscript, also follows a similar flow as Algorithm 1, except that in this approach, each subproblem is solved on the Ising chip. Currently, we perform decomposition on the host, but it is not difficult to move this to hardware in a future version of the chip, as described in Section 4 of this document.

Algorithm 1: Pseudocode of workflow

Input: Input Ising: $F(\mathbf{s}) = \sum_i h_i s_i + \sum_{i=1}^{n} \sum_{j=1, j\neq i}^{n} J_{ij} s_i s_j$, where $i, j \in N$
Output: s
Parameter: *timeout*

Init: **for** *i in N* **do**
    $s_i \leftarrow -1$
end
numIter $\leftarrow 0$
while *numIter* < *timeout* **do**
    $F_{sub}(\mathbf{s}), N_{sub} = Decomposer(F(\mathbf{s}), \mathbf{s})$, where $N_{sub} \subseteq N$
    if *Solve with hardware* **then**
        setup $\leftarrow$ Preprocessing($F_{sub}(s), N_{sub}$)
        $s_i(i \in N_{sub}) \leftarrow$ RunHardware(setup)
    else
        $s_i(i \in N_{sub}) \leftarrow TabuSearch(F_{sub}(\mathbf{s}), N_{sub})$
    end
    $numIter \leftarrow numIter + 1$
    allSAT = checkSolution(**s**))
    if *allSAT* **then**
        Break
    end
end

3 Analysis of Decomposition Methods

Under the same experimental settings in the main paper, Fig. S1 shows the evolution of the All-SAT ratio for the Chancellor^{n+m} formulation across different decomposers. The average all-SAT ratio increases with the number of iterations. The bars depicted in Fig. 5 of the main manuscript provide a concise representation of the curve values at 50, 100, and 500 iterations for all five formulations. Empirically, we see that BFS provides the most All-SAT solutions for the 20-variable benchmarks, followed by SAT decomposer, Pseudorandom decomposer, Random decomposer, and EnergyImpact decomposer. The reason why the Pseudorandom decomposer outperforms the Random decomposer can be explained by the fact that it avoids the repetitive accumulation of the same variable groups in the same subproblem over time. Therefore, Figure S1 serves as a supplementary plot, offering additional insights into the information presented in Fig. 5.

We select the best two formulations (Chancellor^{n+m} and Nüßlein^{n+m}) and use their best decomposer (BFS) to setup our Hybrid 3SAT solver. We use instances 1–10 of uf20-91, which were used in our previous optimization Over 100 iterations and 20 repeats for each benchmark, we plot the average all-SAT ratio in Fig. S2, which shows the results for uf20-91/(1–10). The results for Chancellor^{n+m} are very close to our software-based simulation in Fig. 5, which demonstrates that our Hybrid solver, with the optimizations in Section 5, can solve the 3SAT problem on the Ising chip with similar accuracy as a software-based solver. On the other hand, the average all-SAT ratio for Nüßlein^{n+m} is lower than the software simulation. As reported in the main text, the Chancellor^{n+m} formulation outperforms Nüßlein^{n+m}: the figure shows an improvement in the All-SAT ratio, after 100 iterations, of 36.7% for the chip and 26.8% for the Tabu search solution.

4 Runtime Analysis

Runtime analysis for problem decomposition. The reported runtime analysis does not include the runtime for problem decomposition: we will justify this next by arguing that this can be embedded into hardware at low cost. In

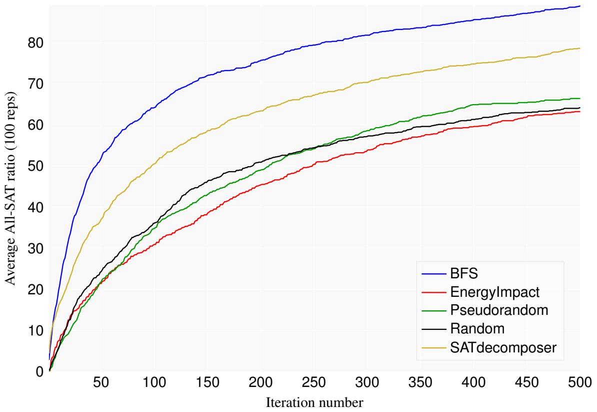

Figure S1. Average All-SAT ratio up to 500 iterations, with 100 repeats, for the first 10 uf20-91 benchmarks using Chancellor^{n+m} formulation.

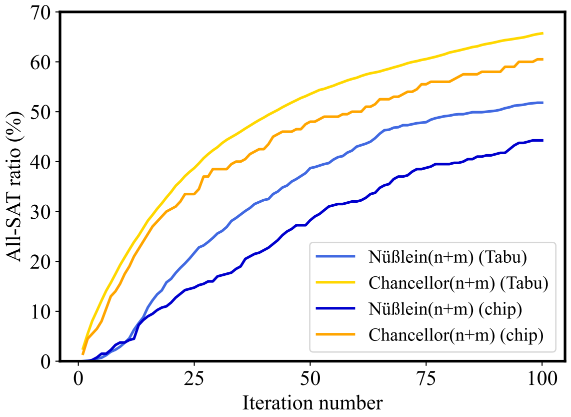

Figure S2. Number of iterations to find All-SAT for hardware test on the Ising chip for Average number of iterations for uf20-91/(01-05, 11-15) for Chancellor^{n+m} and Nüßlein^{n+m} with solver chip and average number of iterations with Tabu search.

general, the runtime for problem decomposition for the next subproblem can be split into four parts:
(1) Generating subproblem indices.
(2) Selecting the coupling weights, J_{ij}, based on these indices.
(3) Updating local fields, h_i, based on the values of "fixed" spins, i.e., spins that are outside the current subproblem and whose values are frozen: a fixed spin s_j changes J_{ij}, s_i, s_j to $\pm J_{ij}$, (depending on whether s_j is $+1$ or -1).
(4) Updating local fields, h_i, based on the values of spins that are updated within the current subproblem.
All of these operations can be performed very efficiently for our hardware substrate. Specifically:
(1) Our random source-based BFS is independent of the iteration and decomposition and can be carried out by precomputing the subproblem indices.
(2) and (3) are not affected by the previous iteration, and require no change from the values precomputed and stored from the previous iteration. We can parallelize them with the Ising solver.
(4) requires updates based on the result of the current subproblem. Implementation of this step on the host is slow and takes hundreds of microseconds.

The use of software-based problem decomposition is only a stop-gap measure because our chip design efforts have been focused on optimizing the computation rather than I/O and operations such as this. All of the above

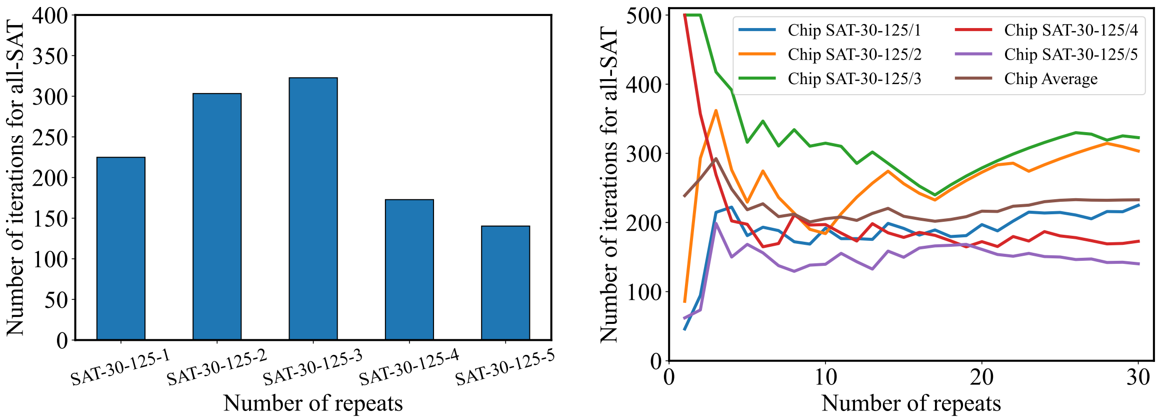

(a) (b)

Figure S3. Number of iterations to find All-SAT for hardware test on the Ising chip for (a) Average number of iterations for 30-125/(1–5) in the formulation of Chancellor^{n+m} with the solver chip. (b) Average number of iterations with repeats for 30-125/(1–5) in the formulation of Chancellor^{n+m} with the solver chip.

computations can easily be implemented on hardware. Currently, digital circuitry on our 65nm chip runs at 125MHz; updating the h_i values for a 45×45 subproblem of a 111×111 global Ising formulation (for a 20 variable 3SAT benchmarks) takes at most a few hundred cycles for the levels of sparsity seen in our formulations (calculation shown below). Therefore, an estimate of runtime on hardware would be a few microseconds, which is very small compared to our IO and RO relaxation time in Figure 7. For this reason, it is neglected.

Calculation of the number of updates: A 48×48 matrix has $48 \times 47/2 = 1128$ off-diagonal elements. Since the density of our coupling matrices is about 10%, this translates to about 113 nonzero J_{ij} values that could contribute to h_i updates. Note that multiple such updates to h_i can be performed within a single clock cycle.

Results on 30-variable problems. Similar to our experiments in Section 6 of the manuscript, we extend our benchmarks to some randomly-generated 30-variable 125-clause 3SAT problems, denoted as benchmarks 1–5 of SAT-30-125. Like the 20-variable benchmarks, these testcases also have a clause-to-variable ratio that is close to the phase transition region[13]. As shown in Fig. S3(a) and (b), over all five benchmarks, the average number of iterations is 232. This is higher than the average of 92 for the 20-variable benchmarks, but our approach is able to solve the problem without too much degradation in runtime over the 20-variable case.

References

1. D-Wave delivers 5000-qubit system; targets quantum advantage (2020). www.hpcwire.com/2020/09/29/d-wave-delivers-5000-qubit-system-targets-quantum-advantage.
2. Sutton, B., Camsari, K. Y., Behin-Aein, B. & Datta, S. Intrinsic optimization using stochastic nanomagnets. *Sci. Rep.* **7**, 1–9 (2017).
3. Debashis, P. *et al.* Experimental demonstration of nanomagnet networks as hardware for Ising computing. In *IEEE International Electron Devices Meeting*, 34.3.1–34.3.4 (2016).
4. Pierangeli, D., Marcucci, G. & Conti, C. Large-scale photonic Ising machine by spatial light modulation. *Phys. Rev. Lett.* **122**, 213902:1–213902:6 (2019).
5. McMahon, P. L. *et al.* A fully programmable 100-spin coherent Ising machine with all-to-all connections. *Science* **354**, 614–617 (2016).
6. Dutta, S. *et al.* Experimental demonstration of phase transition nano-oscillator based Ising machine. In *Proceedings of the IEEE International Electron Device Meeting*, 37.8.1–37.8.4 (2019).
7. Mahboob, I., Okamoto, H. & Yamaguchi, H. An electromechanical Ising Hamiltonian. *Sci. Adv.* **2**, e1600236:1–e1600236:7 (2016).
8. Eslahi, H., Hamilton, T. & Khandelwal, S. Energy-efficient ferroelectric field-effects transistor-based oscillators for neuromorphic system design. *IEEE J. Explor. Solid-St. Comp. Dev. Circ.* **6**, 122–129 (2021).
9. Moy, W. *et al.* A 1,968-node coupled ring oscillator circuit for combinatorial optimization problem solving. *Nat. Electron.* **5**, 310–317 (2022).
10. Takemoto, T., Hayashi, M., Yoshimura, C. & Yamaoka, M. A. A 2×30k spin multichip scalable annealing processor based on a processing-in-memory approach for solving large scale combinatorial optimization problems. In *Proc. ISSCC*, 52–54 (2019).
11. Tanaka, S., Matsuda, Y. & Togawa, N. Theory of Ising machines and a common software platform for Ising machines. In *Proc. ASP-DAC*, 659–666 (2020).
12. Takemoto, T. *et al.* A 144Kb annealing system composed of 9×16Kb annealing processor chips with scalable chip-to-chip connections for large-scale combinatorial optimization problems. In *Proc. ISSCC*, 64–66 (2021).
13. Mitchell, D., Selman, B. & Levesque, H. Hard and easy distributions of SAT problems. In *Proc. AAAI*, 459–465 (1992).
